# Accounting for Viewshed Area and Animal Availability When Estimating Density and Recruitment of Unmarked White‐Tailed Deer

**DOI:** 10.1002/ece3.71015

**Published:** 2025-03-07

**Authors:** Molly M. Koeck, Anna K. Moeller, R. Dwayne Elmore, W. Sue Fairbanks, M. Colter Chitwood

**Affiliations:** ^1^ Department of Natural Resource Ecology and Management Oklahoma State University Stillwater Oklahoma USA; ^2^ Tall Timbers Tallahassee Florida USA

**Keywords:** abundance, camera traps, space‐to‐event, timelapse photography, unmarked population

## Abstract

Quantifying the demography of wildlife is vital to population monitoring; however, studies using physical capture methods can prove challenging. Camera traps have gained popularity as a density estimator tool in recent decades due to noninvasive data collection, reduced labor, cost efficiency, and large‐scale monitoring capabilities. Many wildlife populations are comprised of individuals with no unique natural markers for individual identification, resulting in need for unmarked abundance models. The recently developed Space‐to‐Event (STE) model offers a method for density estimation of unmarked populations using timelapse photography. STE relates detections of animals to camera sampling area (i.e., viewshed), resulting in density estimates that can be extrapolated to abundance over large areas. Consequently, this makes STE sensitive to estimates of viewshed area as small changes in viewshed could significantly affect density estimation. Using STE, we estimated density and recruitment of white‐tailed deer ( *Odocoileus virginianus* ) in a densely forested landscape using measurements of viewshed per camera. We compared estimates of abundance derived from uniquely measured viewshed to estimates of abundance derived from an assumed viewshed area held constant across all cameras. When using a constant viewshed across all cameras, our point estimates of abundance shifted away from uniquely measured viewshed estimates in predictable ways, depending upon how much area was sampled. Additionally, we demonstrated the need for further exploration of animal availability at fine temporal scales by comparing estimates of density derived from sampling the full diel period to estimates derived from periods of peak activity (i.e., crepuscular periods). Finally, we extended the usefulness of the STE model by using densities of fawns and adult females to derive estimates of fawn recruitment.

## Graphical abstract

Monitoring an unmarked population of white‐tailed deer in a densely forested area using camera traps in conjunction with a novel unmarked abundance model. We highlight the importance of unique measurements of camera viewshed area, as well as system closure in abundance estimation through animal availability at fine temporal scales.

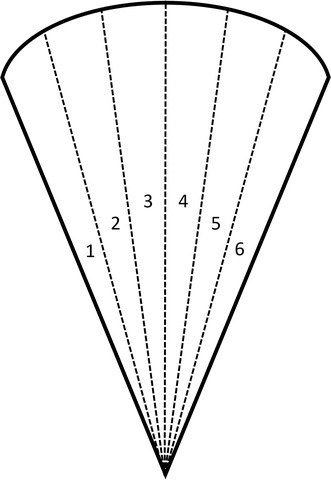

## 1 Introduction

Demographic estimates are vital for managing and monitoring wildlife (Witmer 2005). However, quantifying demography through physical capture can be impractical due to vegetation and terrain. Further, physical capture methods can be invasive and costly (Chandler and Royle 2013). Noninvasive detectors, such as camera traps (hereafter, cameras), are popular as survey tools for abundance estimates (O'Brien 2011) due to their ability to sample across large areas, ease of deployment, and reduced labor (Chitwood et al. 2017). Cameras also allow for the simultaneous capture and monitoring of multiple species with a single camera grid (Tobler et al. 2015), making them a versatile tool for data collection.

Studies monitoring wildlife demographics with cameras often are conducted using capture‐recapture methods of uniquely marked individuals, such as tiger ( *Panthera tigris* ) stripe patterns (Karanth and Nichols 1998) or white‐tailed deer ( *Odocoileus virginianus* ) antler configuration (Jacobson et al. 1997). However, many species are comprised of individuals that cannot be individually identified by unique features, and several models now offer approaches to estimate abundance of unmarked animals. Unmarked abundance models such as the random encounter model (REM; Rowcliffe et al. 2011) and time‐to‐event model (TTE; Moeller et al. 2018) require potentially hard‐to‐obtain auxiliary information on animal movement rate (Bradshaw et al. 2007), while other models may present practical difficulties related to demanding storage and battery life (random encounter and staying time model [REST]; Nakashima et al. 2018) or require particularly dense camera grids (spatial count model [SC]; Chandler and Royle 2013). However, the recently developed space‐to‐event model (STE; Moeller et al. 2018) offers a promising method for estimating abundance of unmarked animals with cameras using timelapse photography in which cameras are triggered on a set schedule rather than by motion detection (Moeller et al. 2018). The STE model is referred to as a “viewshed density estimator” (Moeller et al. 2022) because it relates animal detections to camera sampling area (i.e., viewshed), resulting in density estimates that can be extrapolated to estimates of abundance. When sampling via timelapse photography, the viewshed is equivalent to the viewable area in front of a camera (Moeller et al. 2022), meaning that variable detection inherent in the use of motion triggers is not an issue. Quantifying viewshed area can be achieved by measuring the viewable area at each camera, yielding a unique sampled area that accounts for variation in vegetation structure and terrain among cameras. Because detection via motion sensor is removed from the equation, the STE model does not need to model motion‐sensor variability among cameras (Moeller et al. 2018).

Sampling occasions need to be temporally spaced far enough apart to allow for redistribution of animals across the landscape between sampling occasions (Ausband et al. 2022). At the level of the camera viewshed, animals need to be provided enough time to move in and out of the viewshed prior to the next sampling occasion, as repeated capture of the same individual can bias estimates high. For example, an individual feeding in front of a camera could be detected across multiple sampling occasions and bias the estimates high (similar to how trail‐based or den‐based sampling could bias estimates high). To estimate abundance, STE measures the effort required to observe one individual by randomizing camera order at each sampling occasion and calculating the total area sampled until a detection occurs. However, previous work with STE did not account for variation in vegetation structure or terrain across individual camera viewsheds, potentially leading to biased estimates associated with inaccurate measurements of viewshed. Inaccurate estimation of viewshed area can lead to bias in density estimation when extrapolated to a study area larger than the sampled viewshed area (Moeller et al. 2022). For example, densely forested areas may be particularly sensitive to the use of inaccurate viewshed areas, as vegetation structure varies across the landscape and almost certainly results in different viewable areas from camera to camera. Accounting for visual obstructions can be achieved by dividing the circular sector of the camera lens into multiple sectors and recording the maximum viewable distance per sector, resulting in a unique sampling area per camera (Moeller et al. 2022).

Though the use of STE and other models to estimate abundance from camera trapping data has been explored numerous times recently (Moeller et al. 2018; Loonam et al. 2021; Ausband et al. 2022; Waller 2022; McMurry et al. 2023), extending STE to estimate other demographic parameters is warranted. Recruitment is a useful parameter for assessing population trajectory, and many species can be reliably sorted into juvenile and adult stage classes using camera trapping data. In our context, we define a recruited fawn as one who makes it to the fall hunting season (October 1), which means it has survived the period of greatest mortality over the summer (Cook et al. 1971; Kilgo et al. 2012; Chitwood et al. 2015b). By using photo data on deer detections and separating fawns and females by body size or pelage, fawn‐to‐adult female ratios can be used as a proxy for recruitment. For example, prior work has suggested that camera‐based surveys may be useful to managers for indexing white‐tailed deer fawn (Chitwood et al. 2017) and elk ( *Cervus canadensis* ; Hessami 2019) recruitment using juvenile‐to‐adult female ratios. Moreover, research on various ungulate taxa has demonstrated that fawn survival is more variable compared to adult female survival, suggesting that fawn survival may be the predominant driver of ungulate population dynamics (e.g., elk [Raithel et al. 2007], mule deer [ *Odocoileus hemionus* , Unsworth et al. 1999], white‐tailed deer [Chitwood et al. 2015a], roe deer [ *Capreolus* , Gaillard et al. 1993]). Because juvenile survival is a component of recruitment, extending the use of STE to estimate population size by stage class could be useful for wildlife managers by providing a noninvasive and cost‐efficient survey method for indexing fawn recruitment.

Using white‐tailed deer (hereafter, deer) as a model species, we evaluated STE and the effects of viewshed area in a forested system of Oklahoma. Because deer are easily observable and numerous in southeast Oklahoma (as indicated by annual state wildlife agency harvest reports), we expected to capture ample detections of adults and fawns, allowing us to also explore using STE to estimate recruitment. Additionally, by deploying many cameras, we aimed to evaluate the threshold of cameras that could be used to obtain similar point estimates of density, as well as their levels of precision. By quantifying how viewshed area affects density estimates, extending the STE model to estimate recruitment, and evaluating how few cameras could be used to obtain reasonable estimates, we aimed to provide methods that could be used to estimate demographic parameters of unmarked populations in forests.

### 1.1 Study Area

**FIGURE 1:**
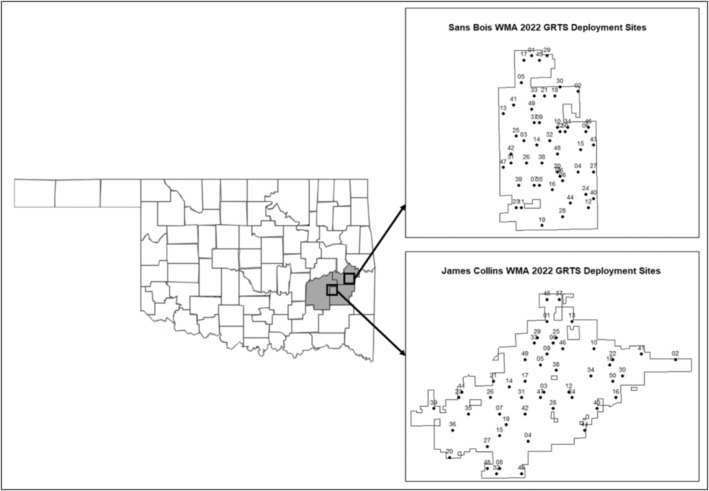
Location of James Collins (JC) and Sans Bois (SB) Wildlife Management Areas (WMA) within three counties (Pittsburg, Latimer, and Haskell) in southeastern Oklahoma. Inset maps show WMA borders with camera trap deployment sites generated using generalized random tessellation stratified (GRTS) sampling for 2021 (*n* = 50 per WMA).

We conducted our study on two sites located in the Ouachita Mountains physiographic region of southeastern Oklahoma: the James Collins and Sans Bois Wildlife Management Areas (WMA; Figure 1). Our sites were owned and managed by the Oklahoma Department of Wildlife Conservation (ODWC) and had considerably different historical forest management (i.e., thinning, prescribed fire), resulting in different vegetation densities and structures that allowed for testing of STE on a variety of forest conditions.

The James Collins Wildlife Management Area (JC) was an 8641‐ha property spanning across Pittsburg and Latimer counties. JC was purchased by ODWC in 1988 and had an extensive history of management (i.e., cattle grazing, eastern redcedar [ *Juniperus virginiana* ] removal, chemical treatment of sericea lespedeza [ *Lespedeza cuneata* ], creation of wildlife food plots, and prescribed fire). An extensive management history, coupled with a well‐established road system (to support extractive energy infrastructure), resulted in a high degree of hunter activity on site. Eastern JC was comprised of ridges and valleys dominated by shortleaf pine ( *Pinus echinata* ) and nonnative loblolly pine ( *P. taeda* ), while western JC was dominated by oak (*Quercus* spp.) and hickory (*Carya* spp.) forests with interspersed native grasslands. ODWC implemented many management strategies on JC, but prescribed fire was used extensively to maintain grasslands and open forest understories. JC bordered the Sans Bois Mountains, a subset of mountains occurring within the larger Ouachita Mountain range, causing a sharp increase in elevation in the southeast region of the WMA. Comparatively, much of the property was comprised of gradual changes in elevation occurring over gentle slopes.

The Sans Bois Wildlife Management Area (SB) was a 3077‐ha property in southeast Haskell County. SB was acquired by ODWC in 2019 and experienced little to no management in the years leading up to its purchase, resulting in intense loblolly and shortleaf pine regeneration and dense understories. Historically, parts of SB were logged for timber, but economic and management efforts were abandoned long before acquisition by ODWC. Occurring entirely in the Sans Bois Mountain Range, SB was characterized as having steep slopes dominated by oak‐pine‐hickory forests and large areas of exposed rock from reclaimed rock mining sites. Densely forested regions of SB had understories comprised of blackberry (*Rubus* spp.), greenbrier (*Smilax* spp.), and loblolly and shortleaf pine saplings. Shortly after purchase, ODWC implemented a prescribed fire program to reduce shortleaf and loblolly pine regeneration and open the forest understory. Elevation varied across the site but peaked at approximately 500 m in the center of the WMA.

## 2 Methods

### 2.1 Camera Deployment

We collected images over 2 years using programmable Reconyx Hyperfire two Professional cameras (Holmen, Wisconsin) capable of timelapse and motion‐triggered photography. We deployed cameras in June of 2021 and 2022 and retrieved them in December of each respective year. Once deployed, cameras synchronously took timelapse photographs to create instantaneous sampling occasions at 10‐min timesteps (i.e., 09:00, 09:10, 09:20, etc.). We selected 10‐min sampling intervals because the time between sampling occasions needs to be long enough for individuals to redistribute relative to viewshed area between occasions to ensure that detections of deer remain independent (Moeller et al. 2018).

Sampling areas of known selection or avoidance can lead to artificially inflated or reduced encounter rates (Rowcliffe et al. 2013), so we used a random sampling approach without the use of any landscape strata or camera distance limitations. By randomly sampling, individuals captured at one camera were not any more or less likely to step in front of the next camera, so we were able to assume independent detections of deer (Moeller et al. 2018). We generated camera deployment sites using generalized random tessellation stratified sampling (GRTS; Stevens Jr. and Olsen 2004; Perret et al. 2022) with the R package “spsurvey” (Dumelle et al. 2023; R Core Team 2023). We produced 50 spatially balanced random points per study site per year (Figure 1), allowing us to evenly sample across a variety of forest structures. GRTS points were re‐generated in 2022 to allow for variation in individual viewsheds.

We affixed cameras to the nearest tree of adequate size to the GRTS‐generated points. We secured all cameras 0.5–1 m off the ground to allow for the simultaneous capture of other species, including smaller‐bodied species. To reduce glare issues associated with direct sunlight on the camera lens, we faced cameras north, unless that direction fully obstructed the viewshed (e.g., facing a large tree trunk or cliff base). We removed potentially obstructing vegetation from the trigger area that was near the camera lens (about 10 m or closer) to reduce the accidental triggering of the motion sensor. Aside from minor vegetation removal, we left the deployment sites minimally disturbed and without bait or lure to reduce attraction or avoidance behavior in target species.

### 2.2 Viewshed Area

We calculated the viewshed area (*a*_*i*_) at camera (*i*) using the circular sector determined by the camera lens angle (_*i*_; 38.72° for Reconyx Hyperfire two cameras) and the maximum viewable distance of detection (*r*_*i*_; Equation 1).

(1) $$a_{i}=\pi r_{i}^{2}\frac{\theta_{i}}{360}$$

**FIGURE 2:**
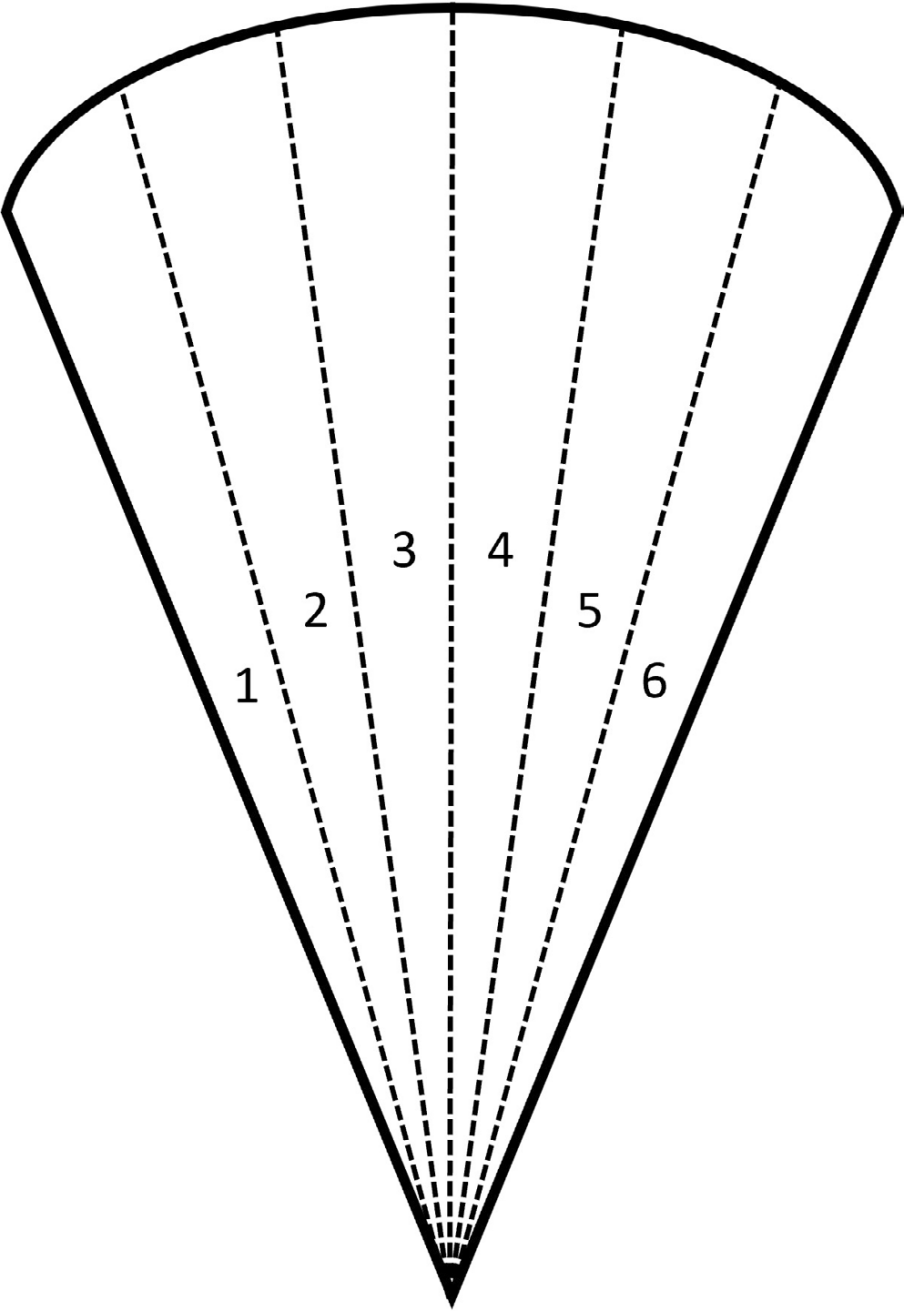
Visual representation of a camera viewshed area (solid lines), divided into six even sectors (dashed lines) for taking maximum distance measurements to account for structural variation across the viewshed.

In densely forested areas, multiple visual obstructions can occur within a small area, creating variation in the maximum viewable distance across a single camera's viewshed and potentially resulting in biased demographic estimates. To account for variation in forest structure, we divided the viewshed area into six even sectors (*j*; Figure 2) and measured the maximum viewable distance per sector (i.e., *r*_*i*_ per sector). We then calculated the viewshed area per sector (*S*_*i*_) and summed the six sectors, resulting in a unique viewshed area per camera (Equation 2).

(2) $$S_{i}=\pi r_{\mathrm{ij}}^{2}\frac{\theta_{\mathrm{ij}}}{360}$$

**FIGURE 3:**
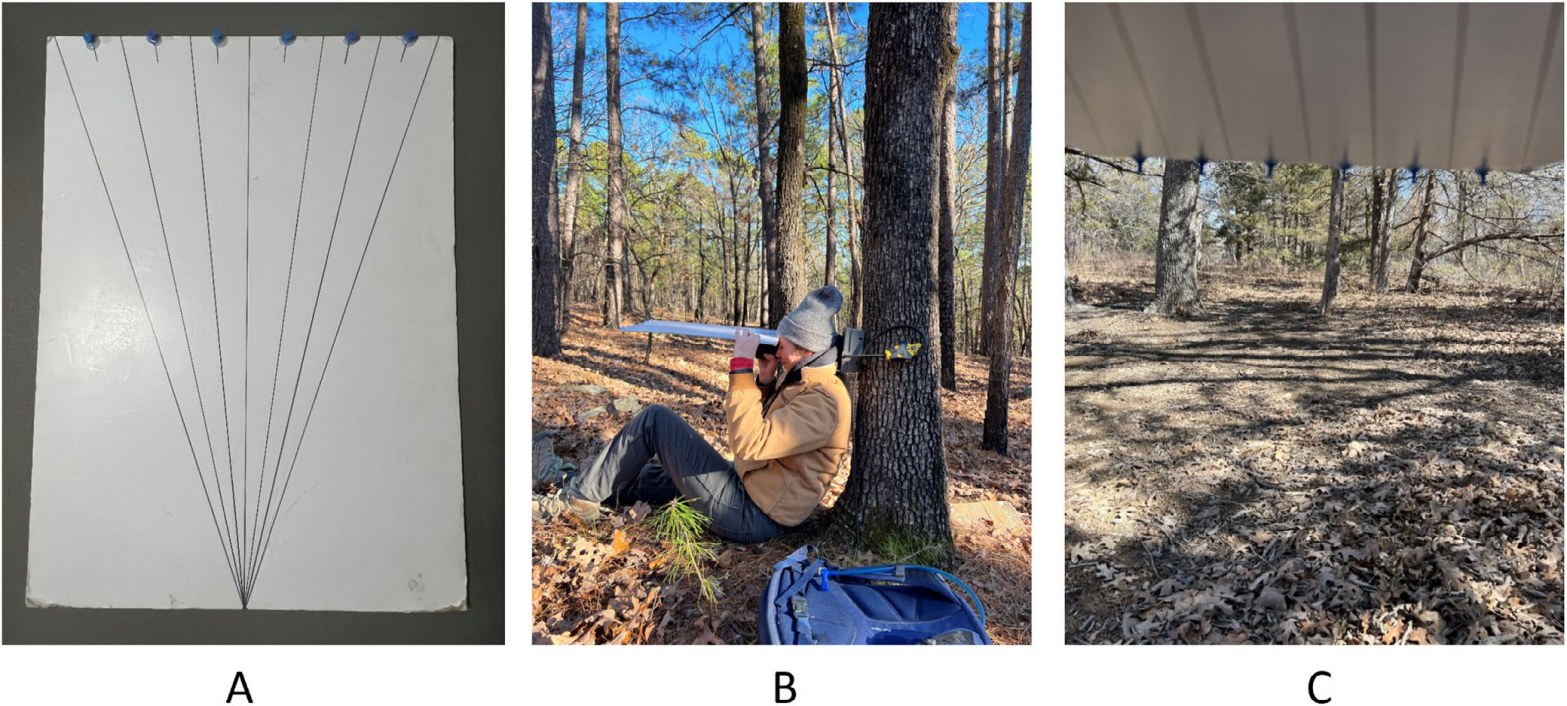
Viewshed board method for measuring camera area in the field. Panel A depicts a viewshed board used for measuring viewshed sectors. Panel B depicts an observer using a viewshed board and laser rangefinder to measure the maximum viewable distance per sector. Panel C depicts the perspective of an observer when measuring the viewshed area with a viewshed board.

At each camera deployment site, we took maximum viewable distance measurements in each sector using a viewshed board (Idaho Department of Fish and Game [IDFG] 2018; Figure 3A) and a laser rangefinder. We constructed viewshed boards by replicating the angle of the camera lens on a 30 × 40‐cm poster board and dividing the angle into six even sectors (6.45 per sector). We recorded viewshed measurements by positioning an observer directly in front of the camera facing the camera's viewshed, such that the observer's eye level was equivalent to the camera lens height. The observer held the viewshed board directly above the eyes (Figure 3B) and parallel to the landscape, such that the six sectors were visible from the observer's perspective below the board (Figure 3C). We applied sight markers (i.e., thumbtacks) to the end of each sector of the viewshed board to help guide the observer's eyes to the location of maximum viewable distance in each sector of the viewshed. After determining the location of maximum distance for a sector, we used a laser rangefinder to record the distance from the camera in meters.

### 2.3 Photo Processing and Analysis

Following camera retrieval, we used Timelapse2 image analyzing software (Greenburg 2023) to tag images and create a database of detections per site and year. We identified all detections to species level (or lowest taxonomic level possible); we further classified deer by stage class (i.e., fawn for individuals < 1 year or adult for individuals > 1 year) and sex (for adults). We used the “spaceNtime” R package (Moeller and Lukacs 2021) to derive STE density and abundance estimates of deer using timelapse photo data collected from 1 August through 30 September; we targeted this period so we could differentiate sex and stage classes in images. We opted to sample prior to the opening day of the Oklahoma deer hunting season (October 1) to avoid behavioral changes in deer due to increased human‐related disturbance associated with hunting. By sampling during late summer, we captured detections of fawns after they became mobile and after the usual spike in fawn mortality at young ages (Cook et al. 1971). We derived deer density from combined detections of all adult male, adult female, fawn, and unknown deer and extrapolated to estimates of abundance. We extended the STE model to estimate recruitment of deer by estimating the density of fawns and adult females separately and calculating the ratio of fawns per adult female.

We initially derived deer density by including all images from the diel period (i.e., 24‐h sampling) to allow for capture of daytime and nighttime detections as deer display varying degrees of nocturnal and diurnal behavior dependent upon season and age (Tomberlin 2007). However, to test for possible biases associated with sampling during times of low activity or inactivity, we subsequently derived estimates of deer density by sampling exclusively during periods of peak activity. Deer are crepuscular and therefore exhibit peak activity near dawn and dusk (Sullivan et al. 2016); thus, we reviewed summaries of detection times at our sites to assess if deer conformed to crepuscularity (i.e., active within 1 h of sunrise or sunset) and removed sampling occasions from time periods where deer did not display peak activity (diurnal and nocturnal occasions). When deer (or stage/sex classes of deer) did not conform to crepuscularity, we derived estimates from periods where peak activity occurred (i.e., diurnal or nocturnal periods). We used the “SunCalc” R package (Thieurmel and Elmarhraoui 2022) to calculate daily sunrise and sunset times associated with the location and date of captures within our sampling frame.

To achieve our objective of assessing the effects of forest structure on density estimation through measurements of viewshed, we compared STE estimates derived from uniquely measured viewshed areas to estimates derived from an assumed viewshed area. We derived STE density estimates of deer using an assumed viewshed area based on camera model manufacturer specifications (i.e., without consideration of the actual landscape characteristics in front of each camera). We used the lens angle and distance of detection of the passive infrared (PIR) motion sensor (30‐m) as a proxy for maximum viewable distance of detection, as many camera brands do not allow for timelapse photography. Thus, absent an independent measure of viewshed distance, users of such cameras would need to truncate by PIR detection distance (at a maximum), as capture would not occur when individuals pass the camera beyond the trigger distance. We calculated the assumed viewshed area and then applied that viewshed area to all cameras to represent sampling without respect to landscape configuration.

### 2.4 Camera Quantity

The STE model assumes independent detections of animals (Moeller et al. 2018) and relies on encounter frequency. Since STE samples the landscape rather than the number of individuals in a population, camera quantity—not camera density—drives precision (Loonam et al. 2021). Camera density differed between our sites, as camera quantity (i.e., 50 cameras) was the same regardless of WMA size. GRTS ensured that we were sampling across our sites in a way that represented the landscape configuration of each WMA. However, in practice, using 50 cameras may preclude the implementation of STE at some sites, so we derived estimates using subsets of our camera data. To achieve our objective of assessing camera precision at varying camera quantities, we estimated deer abundance at increments of 10 cameras (i.e., 10, 20, 30, 40, and 50 cameras) by removing subsets of cameras (e.g., 40 cameras meant the removal of cameras 41–50) so that we obtained one abundance estimate per subset.

## 3 Results

Across 12,200 trap nights (two sampling seasons), we collected 1,662,743 timelapse photos, of which 1252 images were detections of deer (Table 1). In 2021, one camera was stolen from SB, resulting in data being collected across 99 cameras between the two sites; additionally, partial data loss occurred on two cameras at SB due to damage sustained from prescribed fire. In 2022, one camera was stolen from SB and JC each, resulting in data being collected across 98 total cameras.

**TABLE 1:** Summary of total images from data collected August through September of 2021 (‘21) and 2022 (‘22), detections (i.e., images that captured animals), and blanks (i.e., images that did not capture animals), with D representing all white‐tailed deer detections, AF representing adult female deer detections, AM representing adult male deer detections, F representing fawn detections, UD representing unknown deer detections, and O representing other species detections at James Collins (JC) and Sans Bois (SB) Wildlife Management Areas, Oklahoma.

Site year	Total images	D	AF	AM	F	UD	O	Blanks
JC ‘21	415,844	450	231	118	31	125	80	414,809
JC ‘22	407,239	605	453	57	31	94	153	405,846
SB ‘21	417,998	92	66	2	4	23	76	417,735
SB ‘22	421,662	105	56	33	1	17	119	421,331
Total	1,662,743	1252	806	210	67	259	428	1,659,721

### 3.1 Deer Density and Availability

We initially derived density estimates using the diel period, with sampling occasions occurring every 10 min throughout the sampling frame (i.e., 1 August‐30 September; Table 2). After summarizing detection times of deer in our dataset, most deer detections (i.e., peak activity) were during crepuscular time periods (Appendix A1; Table A1). Using crepuscular sampling periods and again using occasions every 10 min, we derived peak activity deer density and abundance estimates (Table 2). We estimated similar densities of deer on SB in 2021 and 2022 when sampling the full diel period; however, we estimated nearly 1.5× the density of deer in 2021 using crepuscular sampling, while 2022 crepuscular estimates were only slightly elevated compared to diel period sampling. In both years, we estimated nearly twice the deer density on JC when sampling crepuscular periods compared to sampling the full diel period.

**TABLE 2:** Density (with standard error [SE] and lower and upper confidence intervals [LCI, UCI]) and abundance (*N*) estimates for white‐tailed deer derived using the space‐to‐event (STE) model. Estimates were derived by sampling the full diel period (Diel) and peak activity period (Peak), where the peak activity period corresponded to crepuscular hours (i.e., +/− 1 h of sunrise and sunset) at James Collins (JC) and Sans Bois (SB) Wildlife Management Areas, Oklahoma, 2021 (‘21) and 2022 (‘22).

Site year	Sampling period	Density (km^−2^)	SE	LCI	UCI	*N*
JC ‘21	Diel	2.28	0.10	1.98	2.40	188
JC ‘22	Diel	4.16	0.17	43.83	4.53	359
SB ‘21	Diel	2.79	0.29	2.27	3.48	86
SB ‘22	Diel	2.34	0.24	1.92	2.86	72
JC ‘21	Peak	3.81	0.35	43.19	4.56	329
JC ‘22	Peak	8.07	0.61	6.97	9.36	698
SB ‘21	Peak	4.32	0.91	2.86	6.53	133
SB ‘22	Peak	2.70	0.65	1.71	4.26	83

### 3.2 Deer Density With Assumed Viewsheds

**FIGURE 4:**
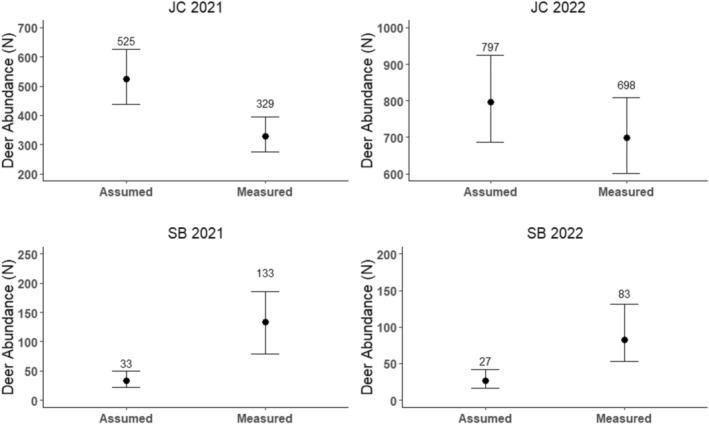
Abundance (N) estimates and 95% confidence intervals for white‐tailed deer across all stage classes and sexes derived using the space‐to‐event (STE) model and peak activity sampling with an assumed viewshed area of 300 m^2^ per camera (Assumed) compared to STE abundance estimates derived from peak activity sampling and using uniquely measured viewshed areas per camera (Measured) for James Collins (JC) and Sans Bois (SB) Wildlife Management Areas, Oklahoma, 2021 and 2022.

Using camera model specifications on the PIR motion detector distance (30 m), we estimated an assumed viewshed area of 300 m^2^ per camera and summed them (*n* = 50 cameras), totaling an assumed sampling area of 15,000 m^2^. We summed camera sampling areas at each site and year to assess differences in area sampled relative to the assumed viewshed area. Total area sampled using unique viewshed measurements ranged from 3741 to 23,671 m^2^ (Appendix A1; Table A2). We used the peak activity sampling parameters (i.e., crepuscular time periods) to estimate deer density with assumed viewshed areas and then extrapolated to abundance (Appendix A1; Table 3). At JC, total area sampled with an assumed viewshed was lower than the total area sampled with uniquely measured viewsheds (Appendix A1; Table A2) and resulting STE estimates were greater when derived using an assumed viewshed (Figure 4). In contrast, at SB, total area sampled with an assumed viewshed was greater than the total area sampled with uniquely measured viewsheds (Appendix A1; Table A2), and resulting STE estimates were lower when derived using an assumed viewshed (Figure 4).

### 3.3 Fawn Recruitment and Availability

As with deer density, we initially derived recruitment (i.e., fawn density divided by adult female density) using the full diel period. In 2021, we derived recruitment rates of 0.15 fawns per female at JC and 0.066 fawns per female at SB. In 2022, we derived recruitment rates of 0.066 fawns per female at JC and 0.021 fawns per female at SB (Table 3).

**TABLE 3:** Density (with standard error [SE] and lower and upper confidence intervals [LCI, UCI]) and abundance (N) estimates for white‐tailed deer fawns and adult females derived using the space‐to‐event (STE) model. Estimates were derived by sampling the full diel period (Diel) and peak activity sampling windows; peak activity corresponded to either Crepuscular (i.e., +/− 1 h of sunrise and sunset) or Diurnal (i.e., beginning 1 h after sunrise and ending 1 h before sunset) periods at James Collins (JC) and Sans Bois (SB) Wildlife Management Areas, Oklahoma, 2021 (‘21) and 2022 (‘22).

Site year	Class	Sampling period	Density (km^−2^)	SE	LCI	UCI	*N*	Recruitment rate
JC ‘21	Fawn	Diel	0.16	0.03	0.12	0.23	14	0.15
	Adult female	Diel	1.10	0.08	0.96	1.26	95	
JC ‘22	Fawn	Diel	0.21	0.04	0.15	0.30	18	0.066
	Adult female	Diel	3.16	0.15	2.87	3.47	273	
SB ‘21	Fawn	Diel	0.13	0.06	0.06	0.32	4	0.066
	Adult female	Diel	1.98	0.25	1.55	2.56	61	
SB ‘22	Fawn	Diel	0.02	0.02	0.00	0.13	0.76	0.021
	Adult female	Diel	1.20	0.17	0.92	1.61	37	
JC ‘21	Fawn	Diurnal	0.31	0.06	0.22	0.45	27	0.17
	Adult female	Crepuscular	1.86	0.24	1.45	2.40	161	
JC ‘22	Fawn	Crepuscular	0.74	0.18	0.46	1.18	64	0.14
	Adult female	Crepuscular	5.46	0.50	4.57	6.53	472	
SB ‘21	Fawn	Diurnal	0.23	0.13	0.07	0.62	7	0.058
	Adult female	Crepuscular	3.93	0.88	2.53	6.04	121	
SB ‘22	Fawn	Diurnal	0.05	0.05	0.01	0.28	1.68	0.033
	Adult female	Crepuscular	1.66	0.49	0.91	2.92	51	

*Note:* Recruitment rate calculated at each site as fawns (ages < 1 year) per adult female (ages > 1 year).

When constraining our estimates to periods of peak activity, we derived estimates of recruitment based on the timing of peak activity for each stage class and year (e.g., JC 2022 diurnal fawn density divided by JC 2022 crepuscular adult female density). Adult female detections indicated crepuscularity; however, peak fawn activity only occurred during crepuscular periods for JC 2021, while fawn activity was greatest during diurnal periods for JC 2021, SB 2021, and SB 2022 (Appendix A1; Table A4). Using peak activity sampling, in 2021, we derived peak activity recruitment rates of 0.17 fawns per female at JC and 0.058 fawns per female at SB. In 2022, we derived peak activity recruitment rates of 0.14 fawns per female at JC and 0.033 fawns per female at SB (Table A3). We noted an increase in recruitment rates after applying peak activity sampling parameters.

### 3.4 Camera Quantity

**FIGURE 5:**
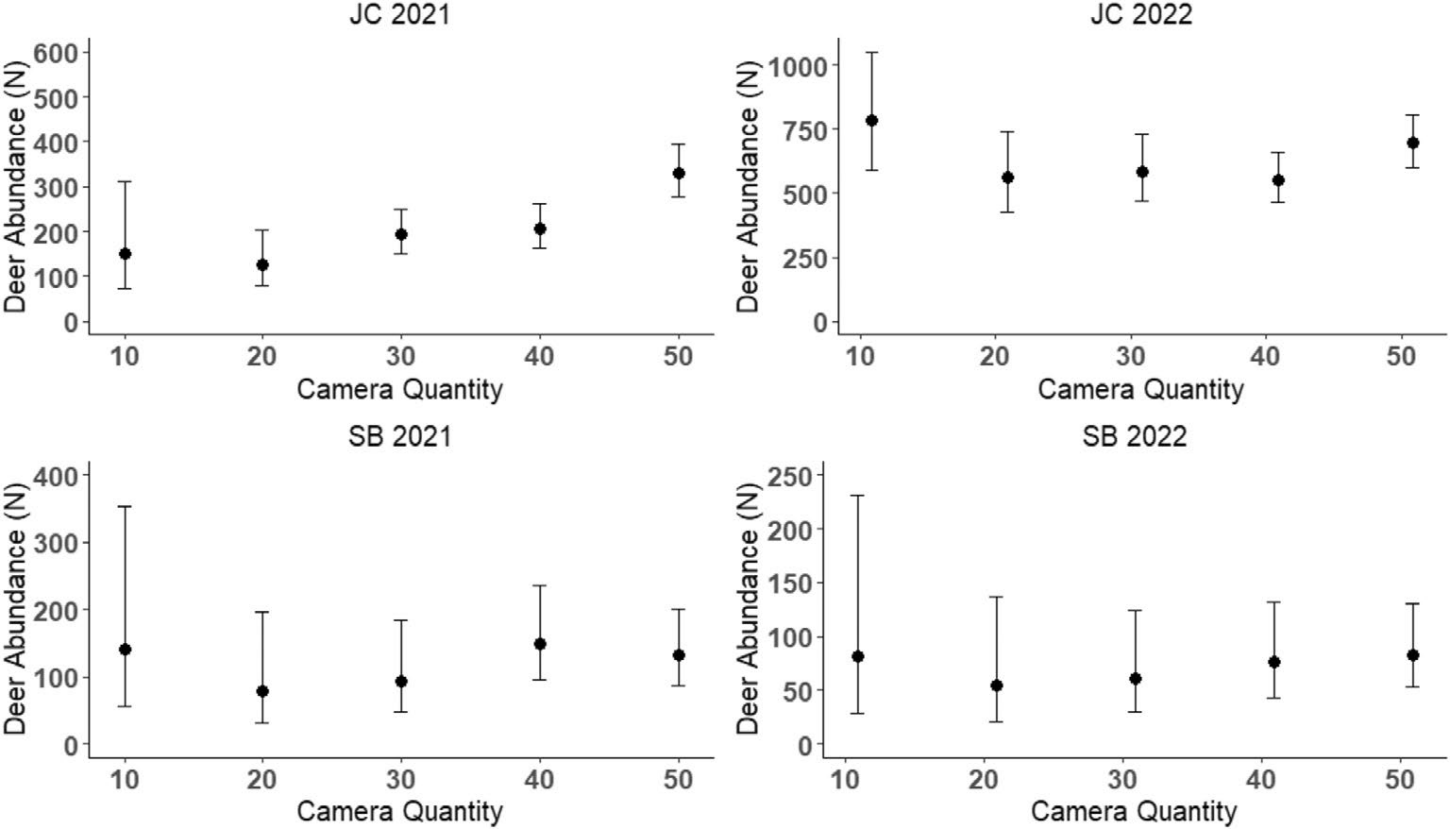
Abundance (N) estimates and 95% confidence intervals for white‐tailed deer across all stage classes and sexes derived using the space‐to‐event (STE) model and peak activity sampling calculated using different camera quantities for James Collins (JC) and Sans Bois (SB) Wildlife Management Areas, 2021 and 2022. Because of camera theft during the deployment period, a camera quantity of 50 is representative of the full camera array, regardless of the number of actual cameras.

We converted estimates of deer density using peak activity sampling to estimates of abundance for comparison at five different camera quantities across sites and years (Figure 5). The number of cameras did not appear to cause major differences in estimates or precision; however, lower precision tended to be associated with fewer cameras (e.g., 10) and greater abundance tended to be associated with more cameras (e.g., 40 or 50; Figure 5).

## 4 Discussion

Recent studies using the STE model for density estimation have produced promising results for several other unmarked species such as gray wolves ( *Canis lupus* , Ausband et al. 2022), cougars ( *Puma concolor* , Loonam et al. 2021), elk (Moeller et al. 2018), wild boar ( *Sus scrofa* , Waller 2022), roe deer (Waller 2022), and sika deer ( *C. nippon* , Waller 2022). Our attempt to extend STE to estimate recruitment provides another population parameter estimate under a single sampling design. Importantly, our study highlighted how animal availability can affect density estimation, potentially hinting at violations of temporal closure at some landscape scales. Additionally, by incorporating viewshed measurements into our density estimates, we demonstrated the effect that assuming the size of the sampling area can have on density estimation.

Our results indicate that camera viewsheds should be individually measured to account for variation in viewsheds across the study area, as forest structure can alter viewshed drastically (e.g., viewshed area across 50 cameras at JC ranged from 9–5627 m^2^ in 2021). Measurements of viewshed have proportional effects on density estimation (Moeller et al. 2022) and mismeasured viewsheds will result in estimates of abundance that are biased high (when sampling area is underestimated) or low (when sampling area is overestimated). By comparing abundance estimates derived from measured and assumed viewsheds (Figure 4), we showed that inaccurate estimates of sampling area have the potential to produce different estimates. More specifically, the direction of effect could differ depending on forest structure at a given site. We suspect the regular prescribed fire regime at JC yielded more open understories and therefore allowed cameras to see farther; therefore, with the measured viewshed, we estimated lower abundance than when we derived the estimate from an assumed viewshed area. By contrast, at SB, where lack of fire yielded dense understories that obstructed camera views, our measured viewshed area was smaller than the assumed area, resulting in greater density estimates. To reduce bias in density estimation, we recommend measuring viewshed, which can be accomplished via viewshed board (this study) or by placing a physical marker in the field at camera deployment and only recording detection within the delineated zone. Our methods were designed to mitigate challenges unique to forested sampling and can be modified when the ecosystem reflects a more open environment. For example, grasslands are more likely to have one single maximum distance measurement spanning the entire viewshed compared to forests, which will have variation in maximum detectable distance across the viewshed. Additionally, modifications to quantifying viewshed area can be made with respect to the targeted taxa (e.g., smaller‐bodied species may not be detectable at great distances so viewshed could be truncated). Moreover, care should be taken when sampling during nocturnal periods, as viewshed area is reduced with lower levels of light (Moeller et al. 2022). With obstructed viewsheds and a dependence on camera flash at night, it may be possible that individuals are present during a nocturnal sampling occasion but go undetected due to their distance from the camera or position relative to obstructions. Although we ultimately excluded nocturnal sampling from our study (see below), we encourage future work on nighttime viewshed measurement strategies to aid in density estimation of nocturnal species.

Because of a related collaborative project, we remeasured viewshed area per camera upon retrieval in December to compare deployment and retrieval datasets on viewshed change over time. By measuring viewshed twice, we discovered data integrity issues with a few summer deployment viewshed measurements that we were subsequently able to correct using December retrieval measurements. Finding these errors (e.g., altered viewshed area post deployment that is not represented by summer measurements) allowed us to correct individual cameras with grossly mismeasured areas that otherwise led to suppressed estimates of density. For example, one camera from the SB 2021 dataset was estimated to observe 8995 m^2^ upon deployment; however, a review of photo data indicated the viewshed was reduced drastically due to vegetation growth shortly after deployment. Indeed, at retrieval, the viewshed area in front of this camera was 157 m^2^, which better represented the viewshed during our targeted sampling window. Just this one difference in area in front of one camera could swing the density estimate at SB for 2021 from 1.17 to 2.89 deer per km^2^. Therefore, we recommend remeasuring viewshed area at retrieval to allow for replacement of mismeasured or altered viewsheds to ensure that the measured area is best representative of the camera viewshed.

The STE model assumes demographic closure and that all animals are available to be detected (Moeller et al. 2018). Demographic closure is usually easy to meet for species with a defined birth pulse by selecting a sampling time when no births occur, and mortality can be assumed to be 0. However, less is known about how animal availability at a finer temporal scale can affect density estimates. In our study, we sampled during a time when the fawn birth pulse was complete and hunting had not yet started, thereby maximizing our ability to meet the assumption of demographic closure. However, within a given diel period, deer could be present in the study area, be off the study area on nearby property, or even be present but unavailable for detection during times of little or no activity (e.g., midday bedding). Indeed, our first density estimates that included data collected for the full diel period were lower than expected based on biologists' knowledge of the study areas (B. Farrar, pers. observation). Upon inspection of our times of detection, we learned that deer were never detected during nocturnal hours via the timelapse dataset, likely due to lower activity coupled with a reduced viewshed area at night. Therefore, we constrained our sampling occasions to time periods when deer demonstrated they were active (i.e., crepuscular period). By focusing on the crepuscular period, we could more safely meet the assumption that all deer were available for detection (Lyet et al. 2023). Resulting density estimates were greater than those derived from the full diel period, and they aligned better with the expectations of local biologists. Though deer do move at night (and were detected using motion sensor), the intersection of timelapse photography and nighttime flash likely caused the lack of nocturnal detections, as both factors can have a negative effect on detection probability. Additionally, the effect of activity and availability on density estimation indicates that other factors could drive underestimates, especially in small or irregularly shaped study areas. For example, biologists knew that some deer at our sites routinely visited feeders on nearby private properties; though those deer did not permanently leave the WMA, they were undetectable to our camera grid at least during the times they visited the feeders, and this lack of closure would drive the density estimate down. Short‐term usage of bait (< 3 weeks) has little effect on spatial variation in deer density, suggesting that home range selection is not affected by short‐term baiting sites (Johnson et al. 2021); however, deer home ranges have been shown to shift with the availability of agricultural crops for prolonged periods of time (VerCauteren and Hyngstrom 1998; Walter et al. 2009). Therefore, long‐term baiting or agricultural practices on neighboring properties could potentially influence deer availability on non‐baited sites, especially in late summer when approaching crop harvest.

The STE model proved effective at estimating density for deer when sampling during periods of high activity, so we used the same approach to derive stage‐specific STE estimates of density that could be used to estimate recruitment. Our time of detection summaries showed that fawns did not always conform to crepuscularity at our study sites. We do not know why fawn activity differed from adult female activity in this manner, but we suspect that some fawns might have been unavailable for detection during certain time periods due to predator avoidance behaviors that resulted in spatial or temporal activity changes (Lima 1998; Thaker et al. 2011). Our study period was designed to minimize such an effect, but we cannot rule it out with our data. Regardless, we opted to derive recruitment rates based on density estimates calculated from peak activity sampling to avoid the likely underestimation problem described earlier. Ultimately, our approach demonstrated that fawn recruitment could be estimated using STE, even when relying on timelapse photography. Most fawn mortality occurs early in life (Cook et al. 1971; Kilgo et al. 2012; Chitwood et al. 2015b), so we assumed fawn survival had stabilized prior to our sampling window. Future work could evaluate biases associated with the timing of sampling, especially if fawn detections are inconsistent due to behavioral shifts in activity or patterns of mortality. Moreover, by deriving recruitment at the same time we estimated density, we increased the value of a single sampling window and camera array.

Camera quantity may be a limiting factor for population monitoring, as WMA managers and biologists may not have access to large quantities of cameras or the resources to purchase and service them. By calculating estimates with subsamples of our 50 cameras, we showed how the total number of cameras used affected the density estimate and its precision. Our results indicated that precision remained consistent at 20 cameras or more. Abundance seemed to increase until 40 or 50 cameras, and those abundances were more consistent with biologist expectations (Figure 5). Therefore, our data indicate that using as many cameras as possible is still the best method for obtaining precise estimates (Moeller et al. 2022); however, there appears to be leeway, at least on smaller, WMA‐sized properties, for reducing the total camera effort and still getting defensible estimates and precision (e.g., 20–30 cameras in this study). A bootstrapping approach can be used to resample STE at varying subsets of camera quantities to derive more robust estimates of density and abundance (Lyet et al. 2023). Additionally, the use of many cameras, coupled with timelapse photography, may result in a large quantity of photos that could also limit the implementation of our methods in management. The use of artificial intelligence (e.g., MegaDetector and Wildlife Insights) could reduce photo‐processing effort required by managers.

Population estimation has the potential to be used for monitoring long‐term population trends and establishing harvest recommendations, so refining methods aimed at reducing biases in density estimation is essential to wildlife population monitoring. STE proved successful in estimating density and recruitment of deer in forested systems. STE assumes demographic closure, so sampling during periods of population stability is vital; however, we suggest constraining sampling to periods of known activity to avoid violating assumptions about availability. Finally, small changes in viewshed area at the level of the camera can drastically alter density estimation, so we suggest using a viewshed board or known marker in the image frame to account for variation within a single viewshed; additionally, repeated measurement of viewshed area upon camera retrieval is one way to account for mismeasured viewsheds or those that were altered since deployment.

## Author Contributions

**Molly M. Koeck:** conceptualization (equal), data curation (lead), formal analysis (lead), investigation (lead), methodology (lead), project administration (lead), visualization (lead), writing – original draft (lead), writing – review and editing (equal). **Anna K. Moeller:** formal analysis (supporting), methodology (supporting), visualization (supporting), writing – original draft (supporting), writing – review and editing (supporting). **R. Dwayne Elmore:** writing – review and editing (supporting). **W. Sue Fairbanks:** writing – review and editing (supporting). **M. Colter Chitwood:** conceptualization (equal), formal analysis (supporting), investigation (supporting), methodology (supporting), project administration (supporting), visualization (supporting), writing – original draft (supporting), writing – review and editing (supporting).

## Conflicts of Interest

The authors declare no conflicts of interest.

## Data Availability

Data has been archived in a Dryad repository at https://doi.org/10.5061/dryad.76hdr7t4g.

## Appendix A1

**TABLE A1:** Summary of total white‐tailed deer detections by time period within a given diel period at James Collins (JC) and Sans Bois (SB) Wildlife Management Areas, Oklahoma, 2021 (‘21) and 2022 (‘22).

Site year	Crepuscular	Diurnal	Nocturnal
JC ‘21	512	457	0
JC ‘22	708	368	0
SB ‘21	228	171	0
SB ‘22	145	116	0

*Note:* Temporal categories were separated into Crepuscular (i.e., +/− 1 h of sunrise and sunset), Diurnal (i.e., beginning 1 h after sunrise and ending 1 h before sunset), and Nocturnal (i.e., beginning 1 h after sunset and ending 1 h before sunrise).

**TABLE A2:** Summed area of uniquely measured viewsheds across all 50 camera traps at James Collins (JC) and Sans Bois (SB) Wildlife Management Areas, Oklahoma, 2021 (‘21) and 2022 (‘22), compared to the total assumed viewshed area using a standardized 300 m^2^ per camera (*n* = 50).

Site year	Area (m^−2^)
JC ‘21	23,671
JC ‘22	20,056
SB ‘21	3,741
SB ‘22	4,769
Assumed viewshed	15,000

**TABLE A3:** Density (with standard error [SE] and lower and upper confidence intervals [LCI, UCI]) and abundance (N) estimates for white‐tailed deer derived using the space‐to‐event (STE) model and peak activity sampling windows and an assumed viewshed area of 300 m^2^ per camera at James Collins (JC) and Sans Bois (SB) Wildlife Management Areas, Oklahoma, 2021 (‘21) and 2022 (‘22).

Site year	Density (km^−2^)	SE	LCI	UCI	*N*
JC ‘21	6.08	0.56	5.08	7.26	525
JC ‘22	9.22	0.69	7.96	10.69	797
SB ‘21	1.07	0.23	0.71	1.62	33
SB ‘22	0.88	0.19	0.55	1.36	27

**TABLE A4:** Summary of the number and timing of white‐tailed deer detections within a given diel period based on stage class (fawns [< 1 year old], or adult female [ages > 1 year old]) at James Collins (JC) and Sans Bois (SB) Wildlife Management Areas, Oklahoma, 2021 (‘21) and 2022 (‘22). Temporal categories were separated into Crepuscular (i.e., +/− 1 h of sunrise and sunset), Diurnal (i.e., beginning 1 h after sunrise and ending 1 h before sunset), and Nocturnal (i.e., beginning 1 h after sunset and ending 1 h before sunrise).

Site year	Class	Crepuscular	Diurnal	Nocturnal
JC ‘21	Fawn	20	47	0
	Adult female	264	232	0
JC ‘22	Fawn	34	11	0
	Adult female	477	227	0
SB ‘21	Fawn	8	11	0
	Adult female	169	114	0
SB ‘22	Fawn	0	22	0
	Adult female	88	87	0

## References

[ece371015-bib-0001] Ausband, D. E. , P. M. Lukacs , M. Hurley , S. Roberts , K. Strickfaden , and A. K. Moeller . 2022. “Estimating Wolf Abundance From Cameras.” Ecosphere 13, no. 2: e3933. 10.1002/ecs2.3933.

[ece371015-bib-0002] Bradshaw, C. J. A. , D. W. Sims , and G. C. Hays . 2007. “Measurement Error Causes Scale‐Dependent Threshold Erosion of Biological Signals in Animal Movement Data.” Ecological Applications 17, no. 2: 628–638. 17489266 10.1890/06-0964

[ece371015-bib-0003] Chandler, R. B. , and J. A. Royle . 2013. “Spatially Explicit Models for Inference About Density in Unmarked or Partially Marked Populations.” Annals of Applied Statistics 7, no. 2: 936–954.

[ece371015-bib-0004] Chitwood, M. C. , M. A. Lashley , J. C. Kilgo , et al. 2017. “Are Camera Surveys Useful for Assessing Recruitment in White‐Tailed Deer?” Wildlife Biology 2017, no. 1: wlb.00178. 10.2981/wlb.00178.

[ece371015-bib-0005] Chitwood, M. C. , M. A. Lashley , J. C. Kilgo , C. E. Moorman , and C. S. Deperno . 2015a. “White‐Tailed Deer Population Dynamics and Adult Female Survival in the Presence of a Novel Predator.” Journal of Wildlife Management 79: 211–219. 10.1002/jwmg.835.

[ece371015-bib-0006] Chitwood, M. C. , M. A. Lashley , J. C. Kilgo , K. H. Pollock , C. E. Moorman , and C. S. Deperno . 2015b. “Do Biological and Bedsite Characteristics Influence Survival of Neonatal White‐Tailed Deer?” PLoS One 10, no. 3: e0119070. 10.1371/journal.pone.0119070. 25734333 PMC4348543

[ece371015-bib-0007] Cook, R. S. , M. White , D. O. Trainer , and W. C. Glazener . 1971. “Mortality of Young White‐Tailed Deer Fawns in South Texas.” Journal of Wildlife Management 35, no. 1: 47–56.

[ece371015-bib-0008] Dumelle, M. , T. Kincaid , A. R. Olsen , and M. Weber . 2023. “Spsurvey: Spatial Sampling Design and Analysis in R.” Journal of Statistical Software 105, no. 3: 1–29. 10.18637/jss.v105.i03. 36798141 PMC9926341

[ece371015-bib-0009] Gaillard, J. M. , D. Delorme , B. Jean‐Marie , G. Van Laere , B. Boisaubert , and R. Pradel . 1993. “Roe Deer Survival Patterns: A Comparative Analysis of Contrasting Populations.” Journal of Animal Ecology 62, no. 4: 778–791.

[ece371015-bib-0010] Greenburg, S. 2023. “Timelapse Image Analyzer and Template Editor.” https://saul.cpsc.ucalgary.ca/timelapse/pmwiki.php?n=Main.Download2. University of Calgary, Calgary, AB, Canada.

[ece371015-bib-0011] Hessami, M. A. 2019. “Estimating Juvenile Recruitment of Elk in an Occupancy Modeling Framework.” Undergraduate Theses, Professional Papers, and Capstone Artifacts, 252.

[ece371015-bib-0013] IDFG . 2018. “Protocol for Statewide Ungulate Camera Deployments.” Idaho Department of Fish and Game, 21.

[ece371015-bib-0014] Jacobson, H. A. , J. C. Kroll , R. W. Browning , B. H. Koerth , and M. H. Conway . 1997. “Infrared‐Triggered Cameras for Censusing White‐Tailed Deer.” Wildlife Society Bulletin 25, no. 2: 547–556.

[ece371015-bib-0015] Johnson, J. T. , R. B. Chandler , L. M. Conner , et al. 2021. “Effects of Bait on Male White‐Tailed Deer Resource Selection.” Animals (Basel) 11, no. 8: 2334. 34438790 10.3390/ani11082334 PMC8388532

[ece371015-bib-0016] Karanth, K. U. , and J. D. Nichols . 1998. “Estimation of Tiger Densities in India Using Photographic Captures and Recaptures.” Ecology 79, no. 8: 2852–2862.

[ece371015-bib-0017] Kilgo, J. C. , H. S. Ray , M. Vukovich , M. J. Goode , and C. Ruth . 2012. “Predation by Coyotes on White‐Tailed Deer Neonates in South Carolina.” Journal of Wildlife Management 76, no. 7: 1420–1430.

[ece371015-bib-0018] Lima, S. L. 1998. “Stress and Decision‐Making Under Risk of Predation: Recent Developments From Behavioral, Reproductive, and Ecological Perspectives.” Advances in the Study of Behavior 27: 215–290. 10.1016/S0065-3454(08)60366-6.

[ece371015-bib-0019] Loonam, K. E. , D. E. Ausband , P. M. Lukacs , M. S. Mitchell , and H. S. Robinson . 2021. “Estimating Abundance of an Unmarked, Low‐Density Species Using Cameras.” Journal of Wildlife Management 85: 87–96. 10.1002/jwmg.21950.

[ece371015-bib-0020] Lyet, A. , S. J. Waller , T. Chambert , et al. 2023. “Estimating Animal Density Using the Space‐To‐Event Model and Bootstrap Resampling With Motion‐Triggered Camera‐Trap Data.” Remote Sensing in Ecology and Conservation 10, no. 2: rse2.361. 10.1002/rse2.361.

[ece371015-bib-0555] McMurry, S. , A. K. Moeller , J. Goerz , and H. S. Robinson . 2023. “Using Space to Event Modeling to Estimate Density of Multiple Species in Northeastern Washington.” Wildlife Society Bulletin 47, no. 1. 10.1002/wsb.1390.

[ece371015-bib-0021] Moeller, A. K. , and P. M. Lukacs . 2021. “spaceNtime: An R Package for Estimating Abundance of Unmarked Animals Using Camera‐Trap Photographs.” Mammalian Biology 102: 581–590. 10.1007/s42991-021-00181-8.

[ece371015-bib-0022] Moeller, A. K. , P. M. Lukacs , and J. S. Horne . 2018. “Three Novel Methods to Estimate Abundance of Unmarked Animals Using Remote Cameras.” Ecosphere 9, no. 8: e02331. 10.1002/ecs2.2331.

[ece371015-bib-0023] Moeller, A. K. , S. J. Waller , N. J. DeCesare , M. C. Chitwood , and P. M. Lukacs . 2022. “Best Practices to Account for Capture Probability and Viewable Area In Camera‐Based Abundance Estimation.” Remote Sensing in Ecology and Conservation 9: 152–164. 10.1002/rse2.300.

[ece371015-bib-0024] Nakashima, Y. , K. Fukasawa , and H. Samejima . 2018. “Estimating Animal Density Without Individual Recognition Using Information Derivable Exclusively From Camera Traps.” Journal of Applied Ecology 55: 735–744. 10.1111/1365-2664.13059.

[ece371015-bib-0025] O'Brien, T. G. 2011. “Abundance, Density and Relative Abundance: A Conceptual Framework.” In Camera Traps in Animal Ecology, edited by A. F. O'Connell , J. D. Nichols , and K. U. Karanth , 71–96. Springer.

[ece371015-bib-0026] Perret, J. , A. Charpentier , R. Pradel , G. Papuga , and A. Besnard . 2022. “Spatially Balanced Sampling Methods Are Always More Precise Than Random Ones for Estimating the Size of Aggregated Populations.” Methods in Ecology and Evolution 13: 2743–2756. 10.1111/2041-210X.14015.

[ece371015-bib-0027] R Core Team . 2023. R: A Language and Environment for Statistical Computing. R Foundation for Statistical Computing.

[ece371015-bib-0028] Raithel, J. D. , M. J. Kauffman , and D. H. Pletscher . 2007. “Impact of Spatial and Temporal Variation in Calf Survival on the Growth of Elk Populations.” Journal of Wildlife Management 71: 795–803. 10.2193/2005-608.

[ece371015-bib-0029] Rowcliffe, J. M. , C. Carbone , P. A. Jansen , R. Kays , and B. Kranstauber . 2011. “Quantifying the Sensitivity of Camera Traps: An Adapted Distance Sampling Approach.” Methods in Ecology and Evolution 2: 464–476. 10.1111/j.2041-210X.2011.00094.x.

[ece371015-bib-0030] Rowcliffe, J. M. , R. Kays , C. Carbone , and P. A. Jansen . 2013. “Clarifying Assumptions Behind the Estimation of Animal Density From Camera Trap Rates.” Journal of Wildlife Management 77: 876. 10.1002/jwmg.533.

[ece371015-bib-0031] Stevens, D. L., Jr. , and A. R. Olsen . 2004. “Spatially Balanced Sampling of Natural Resources.” Journal of the American Statistical Association 99: 262–278.

[ece371015-bib-0032] Sullivan, J. D. , S. S. Ditchkoff , B. A. Collier , C. R. Ruth , and J. B. Raglin . 2016. “Movement With the Moon: White‐Tailed Deer Activity and Solunar Events.” Journal of the Southeastern Association of Fish and Wildlife Agencies 3: 225–232.

[ece371015-bib-0033] Thaker, M. , A. T. Vanak , C. R. Owen , M. B. Ogden , S. M. Niemann , and R. Slotow . 2011. “Minimizing Predation Risk in a Landscape of Multiple Predators: Effects on the Spatial Distribution of African Ungulates.” Ecology 92: 398–407. 21618919 10.1890/10-0126.1

[ece371015-bib-0034] Thieurmel, B. , and A. Elmarhraoui . 2022. Suncalc: Compute Sun Position, Sunlight Phases, Moon Position and Lunar Phase. R Package Version 0.5.1. https://CRAN.R‐project.org/package=suncalc.

[ece371015-bib-0035] Tobler, M. W. , A. Z. Hartley , S. E. Carillo‐Percastegui , and G. V. N. Powell . 2015. “Spatiotemporal Hierarchical Modelling of Species Richness and Occupancy Using Camera Trap Data.” Journal of Applied Ecology 52: 413–421. 10.1111/1365-2664.12399.

[ece371015-bib-0036] Tomberlin, J. W. 2007. “Movement, Activity, and Habitat Use of Adult Male White‐Tailed Deer at Chesapeake Farms, Maryland.” Master's Thesis, North Carolina State University.

[ece371015-bib-0037] Unsworth, J. W. , D. F. Pac , G. C. White , and R. M. Bartmann . 1999. “Mule Deer Survival in Colorado, Idaho and Montana.” Journal of Wildlife Management 63, no. 1: 315–326. 10.2307/3802515.

[ece371015-bib-0038] VerCauteren, K. C. , and S. E. Hyngstrom . 1998. “Effects of Agricultural Activities and Hunting on Home Ranges of Female White‐Tailed Deer.” Journal of Wildlife Management 62, no. 1: 280–285. 10.2307/3802289.

[ece371015-bib-0039] Waller, S. J. 2022. “Evaluating the Use of Camera Traps to Monitor Populations of Ungulate Prey in the Russian Far East.” Master's Thesis, University of Montana.

[ece371015-bib-0040] Walter, W. D. , K. C. VerCauteren , J. M. Gilsdorf , and S. E. Hygnstrom . 2009. “Crop, Native Vegetation, and Biofuels: Response of White‐Tailed Deer to Changing Management Priorities.” Journal of Wildlife Management 73: 339–344. 10.2193/2008-162.

[ece371015-bib-0041] Witmer, G. 2005. “Wildlife Population Monitoring: Some Practical Considerations.” Wildlife Research 32: 259–263. 10.1071/WR04003.

